# Intercellular communication and social behaviors in mycobacteria

**DOI:** 10.3389/fmicb.2022.943278

**Published:** 2022-09-13

**Authors:** Seenivasan Boopathi, Subbiah Ramasamy, B. Haridevamuthu, Raghul Murugan, Maruthanayagam Veerabadhran, Ai-Qun Jia, Jesu Arockiaraj

**Affiliations:** ^1^Key Laboratory of Tropical Biological Resources of Ministry Education, School of Pharmaceutical Sciences, Hainan University, Haikou, China; ^2^Department of Biotechnology, College of Science and Humanities, SRM Institute of Science and Technology, Chennai, Tamil Nadu, India; ^3^Department of Biochemistry, Cardiac Metabolic Disease Laboratory, School of Biological Sciences, Madurai Kamaraj University, Madurai, India; ^4^Biofouling and Biofilm Processes Section, Water and Steam Chemistry Division, Bhabha Atomic Research Centre Facilities, Kalpakkam, Tamil Nadu, India

**Keywords:** quorum sensing, nanotubes, mycobacterial communication, T7SS, biofilm

## Abstract

Cell-to-cell communication is a fundamental process of bacteria to exert communal behaviors. Sputum samples of patients with cystic fibrosis have often been observed with extensive mycobacterial genetic diversity. The emergence of heterogenic mycobacterial populations is observed due to subtle changes in their morphology, gene expression level, and distributive conjugal transfer (DCT). Since each subgroup of mycobacteria has different hetero-resistance, they are refractory against several antibiotics. Such genetically diverse mycobacteria have to communicate with each other to subvert the host immune system. However, it is still a mystery how such heterogeneous strains exhibit synchronous behaviors for the production of quorum sensing (QS) traits, such as biofilms, siderophores, and virulence proteins. Mycobacteria are characterized by division of labor, where distinct sub-clonal populations contribute to the production of QS traits while exchanging complimentary products at the community level. Thus, active mycobacterial cells ensure the persistence of other heterogenic clonal populations through cooperative behaviors. Additionally, mycobacteria are likely to establish communication with neighboring cells in a contact-independent manner through QS signals. Hence, this review is intended to discuss our current knowledge of mycobacterial communication. Understanding mycobacterial communication could provide a promising opportunity to develop drugs to target key pathways of mycobacteria.

## Introduction

Bacteria use a variety of mechanisms to communicate with one another. One of the well-known mechanisms of cell-to-cell communication is QS, in which bacteria secrete, recognize, and respond to chemical messengers with respect to their population density (Mukherjee and Bassler, 2019). However, QS machinery in mycobacteria is still poorly understood. Unlike Gram-positive and Gram-negative bacteria, the cell wall of mycobacteria is rich in lipids, leading to the long-held belief that this rigid lipid layer prevents extracellular ligands/signals from reaching receptor proteins, and thus mycobacteria are considered non-communicating bacteria. However, several experimental pieces of evidence suggest that mycobacteria exhibit QS-related behaviors, such as biofilm, membrane vesicle formation, and horizontal gene transfer (Ojha and Hatfull, 2007; Prados-Rosales et al., 2014; Clark et al., 2018). Cell-to-cell interaction is a prerequisite for the production of QS-related traits. Bacterial interaction may have a significant impact on evolutionary dynamics if interacting bacteria are present at high cell density so that they can perceive each other *via* direct cell-to-cell contact or QS signaling molecules (Nadell et al., 2016). Mice infected with the active *Mycobacterium tuberculosis* (Mtb) strain had about 10,000 bacilli per lung after 10 days, whereas 10 million bacilli per lung were observed after 20 days (Valway et al., 1998), suggesting that mycobacteria exist at high density during active tuberculosis. Such high-density mycobacterial cells exist as filamentous or aggregated forms (Chauhan et al., 2006; Julián et al., 2010) and establish cell-to-cell communication through contact-dependent or -independent mechanism to overcome host-mediated stress conditions through the exchange of molecules. The respiratory tract of patients with cystic fibrosis is infected with multiple pathogens, including different genotypes of *Mycobacterium abscessus, Pseudomonas aeruginosa*, and *Staphylococcus aureus* (Olivier et al., 2003; Levy et al., 2008; Shaw et al., 2019), implying that mycobacteria might interact with different species of bacteria at the niche. Though mycobacteria majorly infect the lungs, multiple genotypes are disseminated to extrapulmonary organs, such as the liver and the spleen (Lieberman et al., 2016). While infecting other parts of the body, such as skin and soft tissue, mycobacteria might interact with native microbiota to compete for spatial stratification and nutrients. However, there are no supporting studies until now to link mycobacterial interaction with other species of bacteria. The coexistence of different species of mycobacteria along with multiple pathogens in the lungs of the patients arguably functions as a polymicrobial pathogenic community, which could be the reason for recurrent treatment failures, as each species and genotypes will have a different range of antibiotic susceptibility. Hence, this review is intended to discuss the recent evidence on mycobacterial communication and how they use social behaviors to promote their persistence in the host.

## Mycobacterial persistence increases with heterogeneity

**Figure 1:**
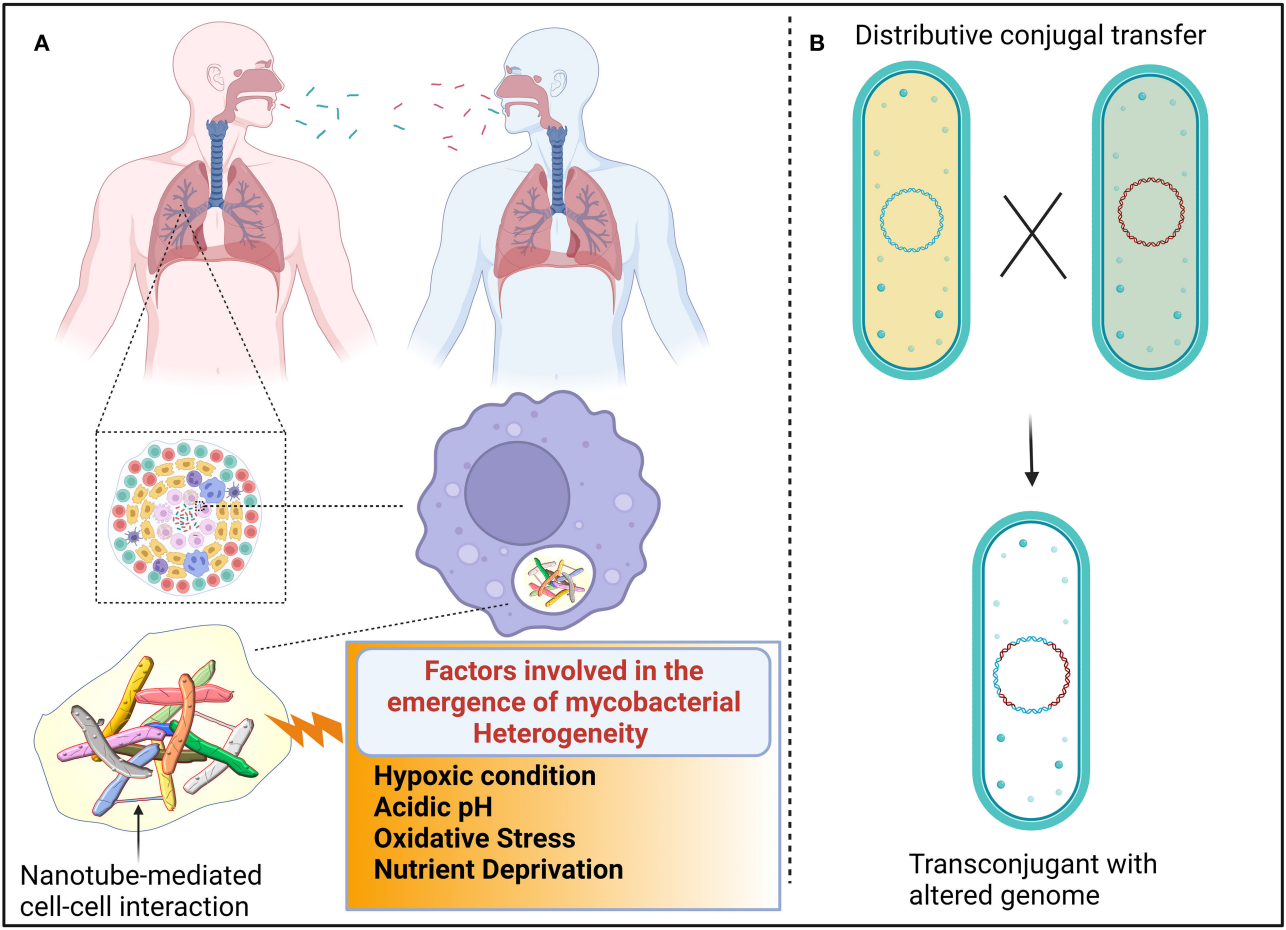
Proposed mechanism for the emergence of heterogenic mycobacteria. **(A)** Different genotypic mycobacteria emerged due to unsynchronized events, such as cell elongation, DNA replication, chromosome segregation, and cell division. Additionally, host immune cells also induce mycobacterial heterogeneity. The composition of granulomatous tissue can vary greatly between individuals, within an individual, and within a single granuloma. The composition and quantity of nutrients and stress-inducing factors are dynamic because each compartment in granuloma shift over time. Such heterogenic mycobacteria are transmitted from one individual to another. Mycobacteria interact with other cells, possibly *via* nanotubes, to exchange quorum sensing traits to establish successful colonization. **(B)** Distributive conjugal transfer (DCT) occurs in mycobacteria wherein multiple segments of DNA from the donor are transferred to the recipient and generate unique genotypes that exert different phenotypic profiles.

Owing to the paucity of advancements in molecular techniques in the last few decades, mycobacteria-mediated lung infection was thought to be caused by a single type of genetically conserved strain. However, using recent advancements in molecular techniques, it was evidently proved that multiple strains of mycobacteria enter the lungs through multiple independent transmission, spatially distributed across different sites of the lungs (Cohen et al., 2012; Lieberman et al., 2016). Interestingly, few reports suggest that Mtb*-*infected patients are also unusually coinfected with non-tuberculosis mycobacteria (NTM) (Huang et al., 2007; Aliyu et al., 2013; Paleiron et al., 2019). NTM causes a broad range of diseases in different parts of the body of immunocompromised patients (Piersimoni and Scarparo, 2009; Misch et al., 2018). The emergence of heterogenic clonal populations among mycobacteria is an inevitable survival strategy that provides fitness to mycobacteria to withstand the hostile host environment. Since factors, such as cell elongation, DNA replication, chromosome segregation, and cell division, are unsynchronized events in mycobacteria, variation in their growth is often observed (Dhar et al., 2016) (Figure 1A). As a result of asymmetrical cell division, mycobacterial cell wall components are unevenly distributed in the dividing daughter cells (Kieser and Rubin, 2014; Hannebelle et al., 2020), resulting in variable responses to antibiotics and host immunity (Dulberger et al., 2020). Empirical evidence revealed that mycobacteria defective to produce LamA have decreased asymmetrical growth and failed to create heterogenic clonal populations (Rego et al., 2017). It was also observed that due to the lack of heterogeneity, cell wall-targeting antibiotics efficiently kill LamA defective mycobacterial cells, implying that cell wall heterogeneity plays an imperative role in fitness. Not surprisingly, the mistranslation of protein also contributes to the emergence of rifampicin resistance in mycobacterial phenotypes under *in vitro* conditions (Javid et al., 2014).

It is adequately represented that the heterogeneity of mycobacteria is also governed by the immune status of the host. Upon sensing the bacterial components, immune cells are assembled at the site of infection, resulting in the formation of lesions. Autopsies of lesions from tuberculosis patients revealed that lesions are heterogeneous in nature in terms of cellular composition and structural organization (Irwin et al., 2015; Carow et al., 2019). As a result, antibiotics penetrate easily in certain lesions, while some lesions receive the least concentration of antibiotics, resulting in the emergence of antibiotic-resistant subsets of bacteria (Prideaux et al., 2015). Mice deficient in the production of interferon γ have less heterogeneous mycobacterial populations than wild-type mice, corroborating that host immune cells also influence mycobacterial physiology (Manina et al., 2015). It could be perceived that heterogeneity among the clonal populations enables them to exhibit multiple personalities to tolerate a variety of stress factors which could result in the persistence of substantial cells even in the presence of multiple attacks (Dulberger et al., 2020). However, cooperation among the heterogenic mycobacterial population is essentially required for successful colonization and survival.

## Cooperation among heterogenic mycobacteria

In mycobacteria, the exchange of genetic material occurs between donor and recipient cells in a systemic manner in which the type VII secretory system plays a vital role throughout the process. The transfer of chromosomal DNA between mycobacteria generates transconjugants with mosaic genomes of the parental strains. During DCT, novel genes or operons from donor bacteria may be inserted into the genomes of transconjugants, or their essential genes may be replaced with the segment of donor bacteria, thus resulting in the change of biochemical pathways in transconjugants, which could influence the fitness of the transconjugants (Gray and Derbyshire, 2018) (Figure 1B). Due to promiscuous DNA replication, daughter cells might lose essential metabolite/protein-encoding genes, which causes them to depend on neighboring cells for nutrients (Boopathi et al., 2021). Though the reciprocal exchange of essential nutrients has been reported in different bacterial pathogens, such molecular trading between mycobacteria has yet not been defined clearly. Metabolic diversification among the clonal population is one of the key strategies for generating heterogeneous subpopulations to survive even in fluctuating nutrient conditions (Simsek and Kim, 2018). Each heterogeneous subpopulation might be specialized in producing certain essential molecules that are required for survival. Therefore, each subpopulation of mycobacteria must depend on each other for their survival. The sub-population of bacteria becomes metabolically inactive, which enables increased fitness in them. Such inactive bacterial cells get benefit from nearby virulence proteins producing active cells without investing in the production of such virulence proteins (Davis and Isberg, 2019). Division of labor may be the common feature of the mycobacterial community, where one subset of cells may produce virulent effectors to subvert host immune systems, while another subset of the population may produce molecules to acquire essential nutrients to benefit the entire population (Davis, 2020). In response to the microenvironments of heterogeneous granuloma, a subset of residing mycobacteria differentially express granuloma-activated genes (Chan et al., 2002), supporting the above-mentioned hypothesis.

### Division of labor

**Figure 2:**
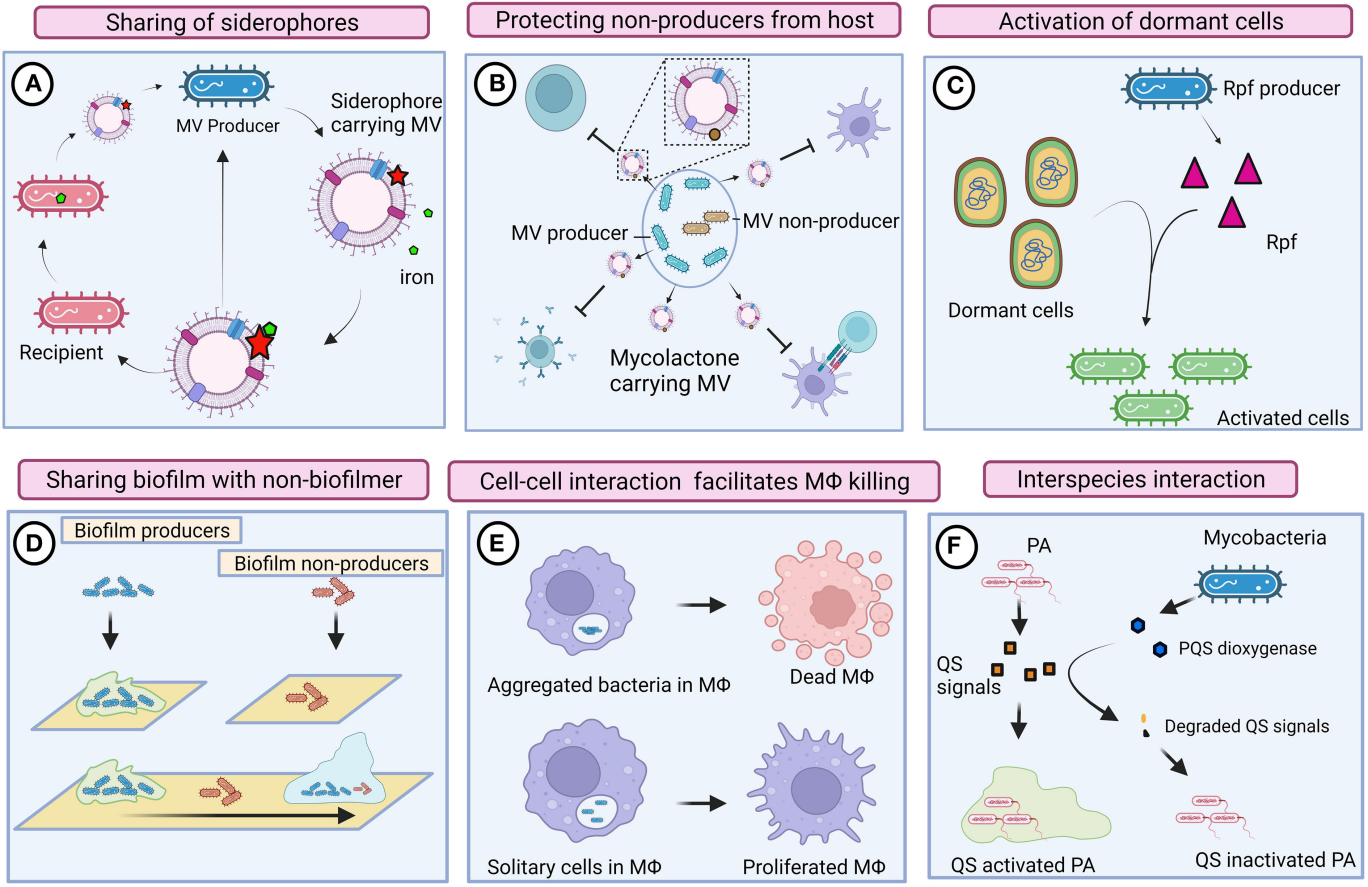
Cooperative and competitive behaviors of mycobacteria. **(A)** Mycobacteria produces mycobactin-carrying membrane vesicles (MVs) to scavenge iron molecules. Siderophore-bound iron enables the growth of mycobacteria that is defective in mycobactin production (Δ*mbtB*). **(B)** Mycobacteria produce mycolactone harboring MV. Mycolactone could protect inactive mycobacterial cells by suppressing the host immune system. **(C)** Resuscitation-promoting factors (Rpf) could reactivate dormant mycobacteria regardless of producers. **(D)** Biofilm-producing mycobacteria could also provide shelter to heterogenic populations (defective to produce biofilm). **(E)** Intrinsic interaction between mycobacteria possibly enables communication and activates macrophage (MΦ) killing pathways. Solitary mycobacterial cells were unable to kill macrophages. **(F)** Mycobacteria compete with *Pseudomonas aeruginosa* (PA) by degrading its quorum sensing (QS) signals. Images are drawn using the Biorender tool (https://Biorender.com/).

Mycobacteria produce three different siderophores, including mycobactin and the soluble carboxymycobactin and exochelin, to efficiently scavenge iron molecules (Dragset et al., 2019). To reduce the metabolic burden, mycobacteria recycle siderophores rather than synthesizing new siderophore molecules (Jones et al., 2014). They also found that the presence of abundant siderophores in both intracellular and extracellular environments is toxic to mycobacteria defective in siderophore recycling. It could be conceived that siderophore recycling in mycobacteria might facilitate them to share siderophores with their local community (Figure 2).

Essentially, membrane vesicles (MVs) also play an imperative role in inter-bacterial communication. Iron-deprived *in vitro* condition induces the production of mycobactin carrying MVs in Mtb, and the mycobactin associated with these MVs serves as an iron chelator and supports the growth of bacteria (Prados-Rosales et al., 2014). MVs from Mtb grown in an iron-deficient medium enable the growth of Mtb defective in mycobactin production (Δ*mbtB*), suggesting that MVs are shared among mycobacteria in the local community (Prados-Rosales et al., 2014). Mycolactone is a toxin required for the development of necrotic cutaneous lesions. Mycolactone harboring MVs have abundantly been found in mycobacterial biofilms (Marsollier et al., 2007), indicating that mycolactone serves as a product of cooperative behavior that also protects other subgroups of bacteria from the host immune system. Another plausible evidence for the cooperative behavior of mycobacteria is resuscitation from a dormant state. Resuscitation-promoting factors (Rpfs) are known to show cell wall hydrolyzing activity and are thus involved in the resuscitation of dormant mycobacterial cells (Nikitushkin et al., 2011). Such cell wall remodeling enzymes are likely to function similar to inter-bacterial signaling enzymes, where Rpfs generate peptidoglycan fragments that act as signaling molecules to activate the resuscitation pathway in dormant mycobacteria (Maitra et al., 2019). Perhaps, resuscitation is not strictly associated with Rpfs producing cells; however, non-producers in the local community also might get benefits by receiving such signals for reactivation.

In response to the relative availability of carbon and nitrogen, mycobacteria either grow as large aggregates or as planktonic cells (DePas et al., 2019). Mycobacteria aggregate in a specific pattern that mimics cords; this phenomenon is known as cord formation. Aggregation, rough morphology, and prolonged persistence of mycobacteria within macrophages have all been found to be strongly correlated (Julián et al., 2010). The rough morphotype of *M. abscessus* forms clumps through tight cell-to-cell interaction that enables them to kill macrophages efficiently, whereas the smooth morphotype remains as isolated bacilli and fails to kill macrophages (Brambilla et al., 2016), suggesting that mycobacterial cells as a community could efficiently subvert the host immune system through intrinsic interaction. Rough (Rg) *Mycobacterium avium* infection caused severe pneumonia and enhanced cytotoxicity in mice and macrophages, respectively (Nishimura et al., 2020). When macrophages were infected with Rg, bacilli were aggregated, and the expression of proinflammatory cytokines, such as TNFα and IL-6, increased. However, Rg could not induce the secretion of proinflammatory cytokines or cause cytotoxicity when they were disaggregated (Nishimura et al., 2020), indicating that intrinsic cell–cell interaction is required for exerting high virulence. Surface-exposed glycopeptidolipids (GPLs) are the outermost components of mycobacteria that mask cell wall-associated lipids. Mycobacteria exhibit rough morphology when they lose several surface-exposed GPLs due to the downregulation or mutation of GPL biosynthetic pathways (Torrelles et al., 2002; Gutiérrez et al., 2018). The absence or reduced expression of GPLs exposes cell wall lipids of mycobacteria and thus induces an immune response. The smooth variant of *Mycobacterium abscessus* is often observed within the phagosome as single bacteria, whereas the Rg variant was found as a group within the phagosome that contains a loose membrane (Roux et al., 2016). Despite the fact that host-induced stress is predominantly prevalent in phagolysosomes, the Rg variant rapidly multiplies and weakens the defense mechanism of macrophages, which results in autophagy and apoptosis (Viljoen et al., 2017), indicating that complex communication between mycobacteria promotes hypervirulence activity. It was also reported that Mtb aggregates might spread *via* bioaerosols (Dinkele et al., 2021). The presence of Mtb aggregates around the lung cavitary lesions of TB patients denotes that hypervirulent Mtb aggregates could transmit from individual to individual and cause increased inflammation and cell death, thus leading to active disease (Rodel et al., 2021). Infection of cells with heat-killed Mtb aggregates did not show significant cell death, suggesting that live mycobacteria intensively communicate with one another and exchange molecular information to elicit virulence within the host.

Mycobacterial biofilms function as a common shelter for cooperative phenotypes in which multiple genotypes, including mycobacteria defective in biofilm formation, are also assimilated, thereby intercellular communication is established between bacterial cells through cell–cell interaction for the exchange of biomolecules (Nguyen et al., 2010). Thus, biofilms also serve as a common shelter for the local community.

### Molecular trading between mycobacteria

Mycobacteria establish communication with neighboring bacteria to exchange multiple fragments of DNA through a unique process of horizontal gene transfer (HGT) that differs from classical *oriT-*mediated conjugation, which provides evolutionary fitness to the bacteria to adapt to a hostile environment (Gray et al., 2013). Prior to the interaction, bacteria must detect suitable partners. Gene knockout studies revealed that type VII secretion systems (ESX systems) are essentially required for inter-bacterial communication (Gray and Derbyshire, 2018). Based on phylogenetic and comparative genomics analysis, it was found that mycobacteria are evolved and harbor different ESX systems in the order of ESX−4, ESX-1, ESX-3, ESX-2, and ESX-5 (Abdallah et al., 2007). Though mycobacteria harbor five T7SS, each ESX system is deserved for unique functions, which implies that one ESX system will not complement other ESX systems due to their distinct secretion signal and differential regulation pathways (Abdallah et al., 2007). Supportively, HGT between mycobacteria happens through the interaction between ESX-1 and ESX-4 systems (Clark et al., 2018), suggesting that interactions between ESX systems are likely to occur to maintain bacterial physiology. It is vital that mycobacterial interaction does not occur between random cells, whereas mycobacteria which possess a mid locus, encoded by *mid* gene, confers mating identity. Transconjugants become donors if the donor-conferring mid-locus also transfers to the recipients (Gray et al., 2013).

Once recipient cells perceive the signal from donor cells, a series of molecular events happen in recipients for the acquisition of molecules. For instance, through unknown signals, once recipient cells recognize donor cells, transmembrane anti-SigM is inactivated, which in turn releases extracytoplasmic transcription factor SigM that activates the ESX-4 system, resulting in the modulation of gene expression that facilitates HGT (Clark et al., 2018). ESX-1 also plays an imperative role in inter-bacterial communication. For instance, donor cells defective in ESX-1 become hyper conjugative (Flint et al., 2004), whereas ESX-1 defective recipient cells do not successfully receive DNA from donors (Coros et al., 2008). Upon interaction with neighboring donor cells, ESX-4-mediated *esxUT* transcripts are upregulated in the recipient cells, whereas such induction of *esx4* transcripts is not observed in the monoculture of either donor or recipient, which gives the notion that ESX-4 is also critically involved in inter-bacterial interaction (Clark et al., 2018). RNA sequencing analysis of coculture revealed that the expression of many other genes was also modulated apart from the genes required for HGT, suggesting that many molecular events occur between donor and recipient cells upon interaction (Gray et al., 2016). Since SigM and ESX-4-mediated signaling system is conserved throughout mycobacteria, such an interaction network might be an active process in different species of mycobacteria. It could be concluded that *esxUT* of recipient cells could be used as a biomarker for intercellular communication (Clark et al., 2018). Molecular trading between bacteria could dramatically alter the mycobacterial molecular pool by directly acquiring foreign molecules, which in turn can influence the colonized niche and infection outcome in the host. Though structural components of ESX-1 similarly exist across different mycobacterial species, their secretory repertoire may be varied between species, even that could be species-specific, which gives the notion that ESX-1 defines the cell surface morphology of mycobacterial cells for cell–cell recognition (Gray and Derbyshire, 2018). Thus, molecular trading in terms of HGT has been well-established in mycobacteria. However, identification of other exchanging molecules could provide deeper insights into mycobacterial physiology.

### Biofilm formation

Mycobacteria can survive in the host for a longer period of time, possibly within the biofilm. Biofilm is a typical organized structure, which harbors a heterogeneous bacterial population that interacts cooperatively to acquire and share resources (Chakraborty and Kumar, 2019). Mycobacteria produce such biofilm in response to environmental cues, such as osmolarity, nutrient availability, pH, oxygen, and CO_2_ concentration (Xiang et al., 2014). Biofilm formation can be viewed as a representation of coordinated behavior, which occurs in both tuberculous and non-tuberculous mycobacteria, including *Mycobacterium tuberculosis, Mycobacterium smegmatis, Mycobacterium marinum, Mycobacterium fortuitum, Mycobacterium chelonae, Mycobacterium ulcerans, M. abscessus, M. avium*, and *Mycobacterium bovis*, affirming that cell–cell communication occurs in mycobacteria (Sharma et al., 2014). Biofilms of non-tuberculous mycobacteria have been reported in different environmental surfaces, including medical devices, dental cavities, membrane filters, and drinking water systems (Schulze-Röbbecke et al., 1995; Falkinham et al., 2015).

Cell–cell interaction is a prerequisite for biofilm formation, where multiple factors critically govern such an interaction. Bacteria effectively communicate with each other if they are present in an aggregated form in the niche. Supportively, rough morphotypic *M. abscessus* makes biofilms that confer resistance against antibiotics (Clary et al., 2018). Lsr2 is a non-specific DNA binding protein, essentially required in donor cells but not in recipient cells for a successful DNA transfer process (Nguyen et al., 2010). Mycobacteria defective in Lsr2 production exhibit altered colony morphology and mutant in biofilm formation (Chen et al., 2006; Arora et al., 2008). Additionally, Mtb Lsr2 regulates the genes involved in adaptation during hypoxia conditions (Bartek et al., 2014), implying that the Lsr2 is a key regulator essentially required for exhibiting multicellular behaviors.

Electron microscopic studies of the lung cavity from the patients infected with *M. abscessus* revealed that mycobacteria exist within the matrix embedded biofilm, suggesting that mycobacteria can efficiently form biofilms under *in vivo* conditions (Qvist et al., 2015; Fennelly et al., 2016). Acid-fast staining of NTM patients' sputum revealed the presence of biofilm-like aggregates (Qvist et al., 2015). It is possible that unsterilized lesions promote the formation of *in vivo* biofilms, contributing to the progression of chronic infection. The lung cavity of the patient harbors a high proportion of mycobacteria along with different species of bacteria (Fennelly et al., 2016), indicating that different strains of Mtb might exist as a community in mixed biofilms *in vivo*. Aggregated mycobacterial cells at the bottom of the liquid media are not able to exchange DNA from donor to recipient, suggesting that biofilm not only serves as a platform to facilitate DNA transfer by enabling cell–cell physical contact but also provides other essential conditions for DNA transfer (Nguyen et al., 2010). Furthermore, molecular evidence is required to elaborate on the mechanism. The emergence of drug-tolerant mycobacteria *in vivo* is possibly due to biofilm formation (Chakraborty and Kumar, 2019). Thus, drug tolerance is a hallmark of the biofilm-resident mycobacterial population. The existence of mycobacteria as a community within the biofilm could be the fact for increased drug tolerance.

Mycobacteria critically govern the mycolic acid production through FAS I and FAS II complexes, where FAS II is involved in the elongation of the FAS I product. FAS II complex consists of six enzymes, including MabA, InhA, KasA, KasB, HadAB, and HadBC, for completing the elongation cycle of fatty acids (Stokas et al., 2020). In addition to FAS I and II, GroEL1 chaperonin plays a significant role in the mycolic acid synthesis and biofilm formation. *M. smegmatis* defective in the *groEL1* gene does not affect the planktonic growth but restricts the formation of mature biofilm and physically interacts with KasA (Ojha et al., 2005). Since the overexpression of GroEL1 protein is positively associated with mature biofilm formation, this protein could be viewed as the hallmark of biofilm formation. Mtb genome consists of 11 serine/threonine kinases (STPKs) that are involved in regulating drug resistance, metabolism, and pathogenicity (Baer et al., 2014). Since STPK phosphorylation regulates the condensation activity of KasA and KasB, changes in the expression of mycobacterial kinase in response to stress conditions may have an impact on the production of mycolic acid (Molle et al., 2006). Furthermore, the role of STPKs in QS traits (e.g., biofilm formation) was demonstrated, stating that overexpression of PknF in *M. smegmatis* resulted in irregular cell structure, as well as defects in sliding motility and biofilm formation (Gopalaswamy et al., 2008). They also discovered that overexpression of PknF alters the composition of glycopeptidolipids, resulting in aggregation. Interestingly, PknF of *M. smegmatis* (PknF_Msm_) does not phosphorylate GroEL1 of *M. smegmatis* (GroEL1_Msm_), but PknF_Msm_ phosphorylates GroEL1 of Mtb (GroEL1_Mtb_) *in vitro*, suggesting that STPKs might involve in the crosstalk between different mycobacterial species (Canova et al., 2009). Mycobacteria lacking functional HadC are less aggregated, exhibit a distinctive mycolic acid profile, have a unique morphology, are deficient in sliding motility and biofilm formation, and are also detergent-resistant (Jamet et al., 2015). HadC mutants are defective in cording formation and exhibit less virulence in the tissue of Mtb-infected mice (Slama et al., 2016). Furthermore, they found that HadC mutants are rifampicin sensitive, implying that mutation or deletion of *hadC* results in virulence loss. Mycobacteria transform from a replication state to a non-replicating persistence state by altering their metabolism and behavior in response to host-induced stress. HadAB and HadBC are downregulated during the stationary phase as a result of STPK-mediated phosphorylation, which suggests that mycobacteria stop producing mycolic acid in a non-replicating condition (Slama et al., 2011). Since cording and biofilm formation are strongly associated with cell-to-cell communication/interaction, HadBC could be a potential target for drug development.

Within the granulomatous lesions, mycobacteria encounter several environmental stresses, including hypoxia, altered pH, nutrient starvation, reactive oxygen and nitrogen species, cell membrane disturbing factors, toxic lipid components, and other cues. To adapt to such a harsh environment, mycobacteria utilize 11 two-component regulatory systems (TCSs) (Bretl et al., 2011). Mycobacteria utilize such TCSs for regulating physiological and metabolic processes, including cell division, stress response, and virulence. The role of TCSs in mycobacterial physiology is extensively reviewed elsewhere (Bretl et al., 2011). This review focuses specifically on the role of TCSs in QS mechanisms and cell-to-cell interaction.

In response to the hypoxic condition in the anaerobic non-replicating persistence (NRP) stage 2 model, it was found that Mtb increases the expression of *fabG, kasB*, and *fbpA* (Gopinath et al., 2015). FabG and Kas are involved in the biofilm formation of mycobacteria (Ojha et al., 2005; Hegde, 2020). Proteins like SigK and DevR are found to be unregulated in both microaerophilic and hypoxic conditions (NRP stage 1 and 2). DevR is one of the components of DosS/DosR that regulate approximately 50 genes during dormancy (Peddireddy et al., 2017). The deletion of the *dosS*/*dosR* regulon from Mtb resulted in less biofilm formation than the wild type (Flores-Valdez et al., 2020), suggesting that DoS/DosR components are crucially involved in the biofilm formation.

## Interspecies interaction

Mycobacteria were thought to be present within granuloma, but they have also been found as extracellular microcolonies outside of granulomatous lesions (Lenaerts et al., 2007). Such extracellular bacteria are recognized as persisters that function as a source for reactivation. Patients with cystic fibrosis are infected with multiple pathogens, where interaction is likely to occur between extracellular bacteria for space and nutrients. *Pseudomonas aeruginosa* and *Aspergillus* spp. were found in great abundance in the sputum samples of CF patients who had been reported to have NTM infections (Levy et al., 2008). Another study found a positive correlation between NTM infection and *Staphylococcus aureus*, but an inverted correlation between NTM and *P. aeruginosa* abundance (Olivier et al., 2003), suggesting the possibility of interspecies communication. During the active phase of tuberculosis, millions of tubercle bacilli are released, which might interact with other bacterial species to subvert the host immune system. Accordingly, they also found that mycobacteria can modulate the pathogenesis of coexisting *P. aeruginosa* and *S. aureus*. For instance, Mycobacteria could influence the QS mechanisms of *Pseudomonas* sp. by degrading their QS signals through PQS dioxygenase and phosphotriesterase-like lactonases (Afriat et al., 2006; Chow et al., 2009; Birmes et al., 2019; Wullich et al., 2019). Likewise, *Mycobacterium fortuitum* modulates the biofilm formation of *P. aeruginosa* by producing pyocyanin demethylase (PodA) that degrades phenazines, thereby inhibiting the pyocyanin-dependent release of extracellular DNA (Costa et al., 2015, 2017). Thus, mycobacteria execute multiple pathways to influence the QS mechanisms of *P. aeruginosa*. Though interspecies interactions are well-defined in other bacterial species (Boopathi et al., 2017a), such interactions are not well-studied in mycobacteria. Moreover, the interaction between slow-growing mycobacteria and fast-growing *P. aeruginosa* is still highly debatable. Further *in vivo* studies are required to support these findings.

## Cell–cell communication

Since HGT occurs in a contact-dependent manner in both fast and slow-growing mycobacteria, it could be conceived that such interaction might prevail across the genus (Gray and Derbyshire, 2018). The constant interaction between mycobacterial cells either on a solid medium or within the biofilm facilitates HGT, whereas HGT does not occur between mobilizing cells (Nguyen et al., 2010). Supportively, HGT is unsuccessful if donor and recipient cells are physically separated using a transwell plate (Gray et al., 2016), denoting that cell–cell contact is essentially required for the exchange of genetic material and for mycobacterial evolution. A previous study has shown that the exchange of genetic material between mycobacteria mechanistically does not happen due to transduction or cell fusion or transformation, suggesting that some specialized mechanisms exist for trading biomolecules between mycobacterial cells (Parsons et al., 1998). However, intercellular communication between mycobacterial species through specialized physical structures is an unexplored phenomenon to date. Since hydrophobic lipid-rich cell wall hinders access to DNA for transformation, it is postulated that HGT occurs in mycobacteria through the specialized structure (Gray and Derbyshire, 2018).

Interestingly, electron microscopy studies suggest that different species of mycobacteria, including *Mycobacterium paratuberculosis, M. avium, Mycobacterium intracellulare, M. bovis, M. tuberculosis, M. marinum, Mycobacterium phlei*, and *Mycobacterium xenopi*, produce hollow extracellular filaments (Draper and Rees, 1970; Merkal et al., 1973; Draper, 1974; Dahl, 2005; Alteri et al., 2007). Furthermore, it was observed that aged mycobacterial cultures produce more extracellular filaments (Dahl, 2005). These structures were referred to by varied nomenclatures as pili, fibrils, or simply extracellular filaments. Despite in-depth insights into the mycobacterial genome and available biochemical and structural data, the functional relevance of these mycobacterial filaments remains elusive. Hence, an in-depth understanding of mycobacterial filaments could provide insights into dimensions, chemical composition, and functionality. Recent molecular evidence reaches certain milestones in deciphering such filaments. For instance, Mtb produces EspC that multimerizes and self-assembles into an extended filament-like structure that is required for the export of EsxA (Ates and Brosch, 2017; Lou et al., 2017). Since ESX-4 tightly regulates cell–cell communication, it could be hypothesized that Esx-4 secreted molecules may function as building blocks to form the tubular structure for the trading of molecules between mycobacteria (Gray and Derbyshire, 2018). Nevertheless, to date, there are no reports on specialized physical structures for establishing communication between mycobacterial strains. Owing to the thick hydrophobic mycomembrane, the mycobacterial cell wall was thought to be highly impermeable, giving the notion that specialized secreting machinery might exist for the transport of molecules (Abdallah et al., 2007).

### Nanotube-mediated communication

Several studies reported that different types of bacteria, including *Bacillus subtilis, Bacillus megaterium, S. aureus*, and *E. coli*, produce nanotubes that form a network between cells for exchanging cytoplasmic molecules, such as amino acids, toxins, proteins, and non-conjugative plasmids, in an intercellular manner (Dubey and Ben-Yehuda, 2011; Pande et al., 2015; Stempler et al., 2017), giving the notion that the above discussed extracellular filaments of mycobacteria are likely to be conduits for transporting cytoplasmic molecules between cells. When neighboring cells are abundantly present in the niche, bacteria establish communication through a network of nanotubes; however, bacteria produce elongated nanotubes in the absence of neighbors (Dubey et al., 2016). In nutrient-deprived conditions, nanotube formation is induced among bacteria to facilitate the movement of nutrients to the intended bacteria (Pande et al., 2015). Supportively, the interaction between auxotroph and donor cells (amino acid producer) *via* nanotubes results in the overproduction of amino acids in donor cells, which is required for auxotroph, thereby deferring feedback inhibition in donor cells (Shitut et al., 2019). Therefore, nanotubes offer selective fitness advantages to bacteria to adapt to the changing environment (D'Souza and Kost, 2016). According to these findings, nanotube formation in bacteria is most likely essential for overcoming nutrient deficiency. Recently, channel-forming membrane integrated proteins (FliO, FlioP, FliQ, FliR, FlhB, and Flh), called CORE, have been found to be essential for nanotube synthesis (Bhattacharya et al., 2019). *In silico* analysis revealed that such CORE complex is widely distributed in different bacterial phyla, including actinobacteria. Since mycobacteria come under the phylum actinobacteria, it could be an indication that mycobacteria are likely to possess such machinery for the synthesis of nanotubes.

Bacteria utilize the cell wall remodeling enzyme LytC and its activator LytB for the extrusion of nanotubes from donor cells and penetration into the recipient cells (Baidya et al., 2020). Mycobacteria are known to harbor several cell wall remodeling enzymes that might be involved in establishing the mycobacterial nanotube network. For instance, *M. smegmatis* has a membrane fission protein called *iniA* that participates in membrane remodeling activities (Wang et al., 2019). Upon physical interaction between mycobacteria, SigM directly regulates the expression of several genes that include ESX-4 apparatus, cell wall hydrolase, and nuclease (Clark et al., 2018), supporting that SigM and ESX-4 pathway might be involved in mycobacterial nanotube extrusion and penetration through the activation of cell wall hydrolase. Upregulation of peptidoglycan amidase (Rv0024) in *M. smegmatis* induces cell–cell interaction that leads to increased biofilm formation, which in turn increases the antibiotic resistance pattern (Padhi et al., 2016), suggesting that cell–cell interaction exists within the mycobacterial biofilm, probably through a nanotube network.

Contact-dependent interaction not only occurs between mycobacterial cells but also between host cells. For instance, Mtb utilizes the ESX-1 system to tear the host cell membrane in a contact-dependent manner (Conrad et al., 2017), which facilitates successful colonization in the host. However, they failed to show tube-like structures that connect bacteria and host cells. However, in a recent study, the mechanism by which host-invaded *E.coli* acquire nutrients from the host cells through nanotube machinery has been demonstrated (Pal et al., 2019). Perceiving messages *via* MVs could be one method of mycobacterial communication. Microscopic analysis revealed that *B. subtilis* is likely to produce MVs from nanotubes through a pinching-off mechanism (Dubey et al., 2016).

### Contact-independent interaction

Although there is sufficient indirect evidence indicating the existence of a possible QS mechanism, no direct evidence has been reported to date in mycobacteria. For instance, a planktonic culture of *M. avium* produces a higher biofilm after the addition of supernatant from biofilm culture, which implies that some QS signaling molecules are present in the supernatant (Carter et al., 2003). C-di-GMP is a secondary messenger that plays an important role in mycobacteria for their survival and adaptation. The C-di-GMP level is determined by diguanylate cyclase (DGC) and phosphodiesterase (PDE-A) (Gupta et al., 2010). As a master regulator, C-di-GMP inter-connected with many regulatory pathways, including QS (Srivastava and Waters, 2012), phosphorylation networks (Lori et al., 2015), and other signal-mediated pathways (An et al., 2013; Gupta et al., 2015). With reference to the protein domain structure, Rv1354c of Mtb is likely to be the crucial component in the C-di-GMP signaling pathway, which may be involved in sensing external signals (Cui et al., 2009). Deletion of C-di-GMP genomic copy in *M. smegmatis* affects its prolonged survival under nutrient-starved conditions, whereas the overexpression of MSDGC-1 (an ortholog of Rv1354c) modulates the morphotypes and growth under *in vitro* conditions (Bharati et al., 2012). When mice are immunized with a BCG strain (ΔBCG1416c) deficient in Delta C-di-GMP diguanylate cyclase, specific CD4^+^ T and CD8^+^ T cells are increased, and host protection is conferred by reducing the burden of Mtb. This finding suggests that C-di-GMP is necessary for mycobacterial survival within the host (Segura-Cerda et al., 2018). Recently, crosstalk between C-di-GMP and two-component regulatory systems is elegantly demonstrated (Hu et al., 2019). They found that when C-di-GMP interacts with DevR, the expression of the *devR* operon increases, allowing mycobacteria to survive under oxidative stress. Thus, the QS mechanism is associated with the DosS/DosR system.

In response to stress or starvation conditions, bacteria produce alarmone (p)ppGpp that regulates several processes, including biofilm, virulence, survival, and persistence (Potrykus and Cashel, 2008). Rel is an alarmone synthetase/hydrolase enzyme that produces (p)ppGpp by hydrolyzing C-di-GMP or transferring pyrophosphate from ATP to GDP (Prusa et al., 2018). When mice are infected with H37RvΔ*rel*_Mtb_, sustained chronic infection is impaired, suggesting that Rel_Mtb_ strongly modulates the expression of components involved in the latent infection (Dahl et al., 2003). Deletion of Rel in *M. smegmatis* resulted in altered cell wall components, decreased long-term survival, and increased vulnerability to hypoxia and nutrient starvation (Dahl et al., 2005). In order to succeed in host-generated stresses, mycobacteria exert several stringent responses that are associated with virulence gene expression, drug tolerance, persistence, and latency. Mtb deficient in Rel_Mtb_ replicates uncontrollably during nutrient deprivation, just like wild-type bacteria, and is unable to enter a dormant state. Mtb lacking Rel_Mtb_ is more sensitive to isoniazid treatment during nutrient starvation and in the lungs of infected mice (Dutta et al., 2019). Therefore, (p)ppGpp homeostasis is required during chronic infection in animals to regulate metabolism and virulence in the face of host-mediated stress.

Another indirect evidence also has speculated the presence of the QS system in mycobacteria. It showed that a putative transcriptional regulator's (*whiB3*) expression relied on bacterial density, reflecting the possible existence of the QS mechanism (Banaiee et al., 2006). Mycobacteria overcome host-mediated stress through the use of Whib3, which reprogrammes metabolic activity in response to oxidative, reductive, and nitrosative stresses. Through comparative genomics study, numerous proteins in Mtb are found to be associated with QS mechanisms (Hegde, 2020). The LuxR transcriptional regulator is a component of the QS circuit, which controls the expression of several genes, including those encoding bioluminescence, virulence factors, and biofilm formation. Sequencing analysis revealed that seven LuxR family proteins, such as Rv0386, Rv0195, Rv0491, Rv0890c, Rv0894, Rv2488c, and Rv3133c, are found in the genome of Mtb (Chen and Xie, 2011). A QS signal, such as autoinducer-2 (AI-2), induces oxidative stress which in turn induces biofilm formation in *M. avium* (Geier et al., 2008), suggesting that AI-2 could be recognized by mycobacteria which allow downstream QS events. *In silico* analysis has revealed that Mtb possesses a gene that has 59% homology and 41% identity with LuxS, which is responsible for AI-2 production (Surette et al., 1999). Like AI-2, diffusible signal factor (DSF) activity was detected in different mycobacterial species, including *M. avium, M. chelonae, M. smegmatis, M. intracellulare*, and *Mycobacterium kansasii* (Wang et al., 2004). Thus, mycobacteria presumably execute contact-dependent and contact-independent interactions concurrently.

## Conclusion and future directions

In light of the findings of several reports, it can be concluded that mycobacteria also execute inter-bacterial communication. Unambiguously, mycobacteria also detect and respond to the neighboring cells that could be either their clone mates or other bacterial species. Despite the existence of a heterogeneous population, mycobacteria exhibit multicellular behavior under *in vivo* conditions, in which each subset of cells divides their work themselves (a division of labor) rather than producing all essential molecules to reduce their metabolic burden. The fact is that cell-to-cell communication is an integral part of the evolution of mycobacteria and the increase of rich diversity. While considering the importance of inter-bacterial communication for the exchange of genetic material, many fascinating questions are also raised. Whether mycobacteria exchange any other cytoplasmic molecules, such as proteins, RNAs, toxins, nutrients, and plasmids, with the neighboring cells? The existence of multiple subsets of the mycobacterial population at the site of infection, intriguing to speculate that one subset of cells confers resistance to certain antibiotics, while other subsets of cells produce some other antibiotic resistant-conferring proteins. The frequent incidence of failures in antibiotic therapy might be due to the exchange of those resistance-conferring molecules among the population. However, the coexistence of both subtypes benefits the entire population during antibiotic treatment. The exchange of antibiotic resistance-conferring proteins/mRNAs between different genotypes of *B.subtilis* has been well-documented (Dubey and Ben-Yehuda, 2011). The response of mycobacteria to AI-2 suggests the presence of common QS mechanisms across the mycobacterial species that might control several vital processes in mycobacteria.

Since virulence proteins are crucial during infection, it is imperative to find the factors that control/regulate the expression of these proteins. Hence, understanding the regulatory pathways could give insights into the molecular nature of mycobacterial infection (i.e., tuberculosis and leprosy). Key regulatory proteins of QS mechanisms could be used as a target to control pathogenesis (Boopathi et al., 2022b). As mentioned before, key proteins that are conserved across mycobacteria may be involved in QS mechanisms of various mycobacterial species, which could be targeted as a common target to control various mycobacterial diseases. Analogs of QS signals could be used as competitive inhibitors to attenuate the QS mechanisms (Boopathi et al., 2017b, 2022a), thereby mycobacterial virulence could be inhibited during pathogenesis. Moreover, identifying drugs that specifically target key regulatory proteins of QS mechanisms could be the potential approach for drug development.

## Author contributions

SB designed the overall idea and wrote the manuscript. RM and BH assisted in the artwork. SR, MV, A-QJ, and JA performed the review of the literature, reviewed, and corrected the manuscript. All authors contributed to the article and approved the submitted version.

## Funding

This work was supported by the National Natural Science Foundation of China (82160664).

## Conflict of interest

The authors declare that the research was conducted in the absence of any commercial or financial relationships that could be construed as a potential conflict of interest.

## Publisher's note

All claims expressed in this article are solely those of the authors and do not necessarily represent those of their affiliated organizations, or those of the publisher, the editors and the reviewers. Any product that may be evaluated in this article, or claim that may be made by its manufacturer, is not guaranteed or endorsed by the publisher.
